# Supplementary material for: Perinatal hormones favor CC17 group B Streptococcus intestinal translocation through M cells and hypervirulence in neonates
Source: eLife. 2019 Nov 11;8:e48772. doi: 10.7554/eLife.48772 (PMC6867712; doi:10.7554/eLife.48772)
Supplement: Supplementary file 1. [file elife-48772-supp1.docx]

**Supplemental Table. Primers used for RT-qPCR**

|  | Sequence (5’-3’) |
| --- | --- |
| **RT-qPCR - Mouse genes** | |
| *Actin* | F: TCCTCCTGAGCGCAAGTACTCT  R: CGGACTCATCGTACTCCTGCTT |
| *Ccl19* | F: ATGTGAATCACTCTGGCCCAGGAA  R: AAGCGGCTTTATTGGAAGCTCTGC |
| *Ccl20* | F: AAAAGGGCTGTGAACCTCCT  R: ACCCCAGCTGTGATCATTTC |
| *Ccl21* | F: TGAGCTATGTGCAAACCCTGAGGA  R: TGAGGGCTGTGTCTGTTCAGTTCT |
| *Ccr6* | F: CCTCACATTCTTAGGACTGGAGC  R: GGCAATCAGAGCTCTCGGA |
| *Ccr7* | F: TCATTGCCGTGGTGGTAGTCTTCA  R: ATGTTGAGCTGCTTGCTGGTTTCG |
| *Rankl* | F: TGTACTTTCGAGCGCAGATG  R: ACATCCAACCATGAGCCTTC |
| *Spib* | F: AGCGCATGACGTATCAGAAGC  R: GGAATCCTATACACGGCACAGG |
| *Gp2* | F: GATACTGCACAGACCCCTCCA  R: GCAGTTCCGGTCATTGAGGTA |
| **RT-qPCR - Human genes** | |
| *ACTIN* | F: AGAGCTACGAGCTGCCTGAC  R: TGATCTTGATCTTCATTGTG |
| *RANKL* | F: TGGTTCCCATAAAGTGAGTC  R: GAAGATACTCTGTAGCTAGGTC |
| *SPIB* | F: GAGGAAGACTTACCGTTGGACAG  R: TCAGTAGCCCCAGCAGGAA |
| *TNFAIP2* | F: ATCGACATCATCCAGATCAC  R: TAACATTGGCCCTGTAATTC |
| *GP2* | F: GAGAGAAATCAAACCCATGCC  R: ACTGCATCCCCTTCGTAAGGA |
